# Depotentiation from Potentiated Synaptic Strength in a Tristable System of Coupled Phosphatase and Kinase

**DOI:** 10.3389/fncom.2016.00104

**Published:** 2016-10-19

**Authors:** Mengjiao Chen, Wei Ren, Xingang Wang

**Affiliations:** ^1^School of Physics and Information Technology, Shaanxi Normal UniversityXi'an, China; ^2^Key Laboratory of Modern Teaching Technology, Ministry of Education, Shaanxi Normal UniversityXi'an, China

**Keywords:** long-term potentiation, depotentiation, tristability, kinase, phosphatase

## Abstract

Long-term potentiation (LTP) of synaptic strength is strongly implicated in learning and memory. On the other hand, depotentiation, the reversal of synaptic strength from potentiated LTP state to the pre-LTP level, is required in extinction of the obsolete memory. A generic tristable system, which couples the phosphatase and kinase switches, exclusively explains how moderate and high elevation of intracellular calcium concentration triggers long-term depression (LTD) and LTP, respectively. The present study, introducing calcium influx and calcium release from internal store into the tristable system, further show that significant elevation of cytoplasmic calcium concentration switches activation of both kinase and phosphatase to their basal states, thereby depotentiate the synaptic strength. A phase-plane analysis of the combined model was employed to explain the previously reported depotentiation in experiments and predict a threshold-like effect with calcium concentration. The results not only reveal a mechanism of NMDAR- and mGluR-dependent depotentiation, but also predict further experiments about the role of internal calcium store in induction of depotentiation and extinction of established memories.

## Introduction

Over the past decades, ample evidences support that long-lasting modifications of synaptic strength, including long-term potentiation (LTP) and long-term depression (LTD), are strongly implicated in learning and memory. Meanwhile, depotentiation, which refers to reversal of synaptic strength from potentiated LTP state to its pre-LTP level, is required by extinction of the obsolete memory. In the amygdala, cued fear-conditioning induces widespread synaptic strengthening (McKernan and Shinnick-Gallagher, 1997; Rogan et al., 1997; Rumpel et al., 2005). There is growing evidence to support that fear memories are transiently susceptible to erasure due to the ability of the same experience to reverse this form of synaptic strengthening (Kim et al., 2007; Clem and Huganir, 2010; Díaz-Mataix et al., 2011). A similar role of depotentiation is also found in hippocampal-dependent memory extinction (Zhang et al., 2011; Wang and Zhang, 2012). Novelty acquisition is not only able to induce LTP (Li et al., 2003; Gruart et al., 2006; Whitlock et al., 2006; Madroñal et al., 2007), but also enables induction of depotentiation from previously established LTP (Xu et al., 1998; Manahan-Vaughan and Braunewell, 1999; Abraham et al., 2002; Straube et al., 2003; Collingridge et al., 2010). These findings indicate that experience or stimulation protocol resulting in LTP can further induce depotentiation. However, mechanisms for depotentiation induced by further application of stimulations for LTP induction still remain unclear.

There have been a number of substantial theoretical investigations of the multi-stable mechanism of phosphatase and kinase switches which could account for LTP and LTD. Early simulation works proposed that the bistable switch formed by kinase, Ca^2+^/CaM protein kinase II (CaMKII), autophosphorylation underlies the stable transition between the basal and LTP states (Lisman, 1989). Recently, the authors further show that autodephosphorylation of protein phosphatase 2A (PP2A), one of the few serine/threonine-specific phosphatases, also forms a bistable switch, which could account for the stable transition between the basal and LTD states. Even more importantly, coupling of the phosphatase and kinase switches produces a tristable system, which exclusively explains how moderate and high elevation of intracellular calcium concentration triggers LTD and LTP, respectively. A phase-plane analysis was employed to understand the model graphically, in which nullclines at different calcium concentrations were plotted to show how the system moves during LTD and LTP induction and how it is finally stabilized after the induction, respectively (Pi and Lisman, 2008). This tristable system provides a generic theoretical framework for understanding dynamic mechanisms for LTP and LTD. Depotentiation induced by LTP induction protocol from the established LTP state to its basal state, which may be triggered by even higher elevation of intracellular concentration, also needs to be studied theoretically.

In a number of experiments, it has been shown that biochemical machinery for depotentiation is also formed by the molecular processes responsible for LTP, including calcium influxes, activation of specific enzymes, and glutamate receptor trafficking. Blockage of depotentiation when the induction stimulation was delivered in the presence of an antagonist of N-methyl-D-aspartic acid (NMDA) receptors (NMDARs) suggests that activation of NMDA receptors is required (Christie et al., 1995; Abraham and Huggett, 1997). Activation of metabotropic glutamate receptors (mGluRs) has also been shown required for depotentiation (Bikbaev et al., 2008; Qi et al., 2013). Besides membrane permeability (activation of Ca^2+^ channels), the amount of Ca^2+^ influx depends on the calcium ion concentration difference in the intracellular vs. extracellular spaces, which causes diffusion of calcium ion from high to low concentrations. So, in experiment, lowering extracellular calcium ion is applied as one way to reduce influx and intracellular calcium ion elevation. Stimulation is no longer able to induce depotentiation when it is delivered in a condition in which extracellular Ca^2+^ concentration is lower than normal (Abraham and Huggett, 1997), suggesting elevation of intracellular Ca^2+^ concentration is also required. In addition, phosphatase inhibitor can block depotentiation, showing involvement of protein phosphatase pathways in depotentiation induction (Kang-Park et al., 2003). The above experimentations strongly suggest that, after the establishment of LTP, further increase of intracellular calcium concentration, which could be initiated by calcium influxes mediated by NMDARs and mGluRs, may regulate phosphatase and kinase pathways and then switch synaptic strength back to its pre-LTP level.

In the present study, calcium influxes through NMDARs and calcium release from internal calcium stores by activation of mGluRs are introduced to the tristable system to simulate the experimentally observed depotentiation by using LTP-induction protocol. An phase-plane analysis of the dynamics for transition from LTP to its basal level is also provided.

## Model and methods

**Figure 1:**
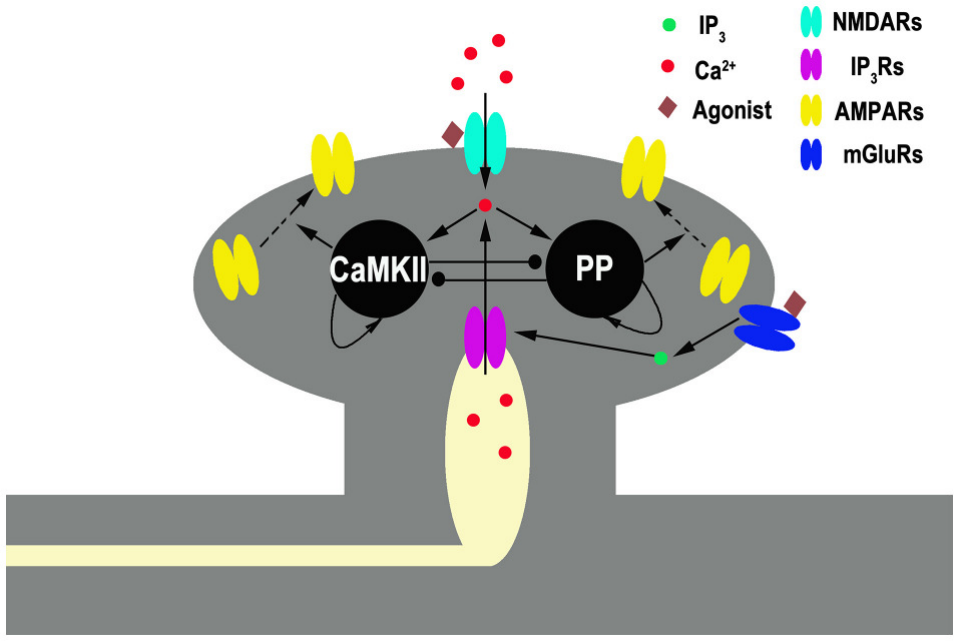
**Schematic diagram of the full model**. Gray shading represents spine structure and light yellow shading represents internal stores [endoplasmic reticulum (ER)]. Pointed and circled arrows indicate the activation and the inhibition pathways, respectively. An agonist such as neurotransmitter binds to NMDARs and mGluRs. The latter one initiates a series of reactions, linked through G-protein, that ends in the production of the second messenger inositol trisphosphate (*IP*_3_), which diffuses through the cytoplasm and binds to *IP*_3_ receptors (IP_3_Rs) locating at ER membrane. IP_3_Rs are calcium channels binding with *IP3* and mediating calcium release from the ER into cytoplasm. NMDARs are membrane calcium channels binding with glutamate mediating calcium influx. Cytoplasmic Ca^2+^ activates CaMKII (K) and protein phosphatase (P) in a manner that depends on the concentration of cytoplasmic Ca^2+^. CaMKII and phosphatase can self-activate themselves and inhibit each other. Active kinase and phosphatase control AMPARs insertion and removal, respectively.

Our model contains two primary ingredients as schematized in Figure 1. The first is a mechanism through which the plasticity of synaptic strength can be depicted. Here, we adopt the tristable biochemical network described by Pi and Lisman (2008), which describes coupling of the CaMKII switch and PP2A switch in the postsynaptic site of synapses. The system yields three different stable states, corresponding to the basal, the LTP, and the LTD levels of synaptic strength, respectively. Transient moderate elevation of calcium concentration can induce LTD, while high elevation induces LTP. In this model, the values of phosphorylated CaMKII and dephosphorylated PP2A govern α-amino-3-hydroxy-5-methyl-4-isoxazole-propionic acid (AMPA) receptors (AMPARs) trafficking which determines the number of AMPAR inserted in the postsynaptic membrane and hence the synaptic efficacy. A basal state (with basal AMPARs level) is determined by low concentrations of both phosphorylated CaMKII and dephosphorylated PP2A, a LTP state (with high AMPARs level) by high phosphorylated CaMKII and low dephosphorylated PP2A, and a LTD state (with low AMPARs level) by low phosphorylated CaMKII and high dephosphorylated PP2A, respectively. Switching between these states is controlled by cytoplasmic calcium concentration in the post synaptic site. These states are self-sustaining; once the system moves into one of these states, it remains there, although calcium concentration returns to the basal level.

The second key part of our model is a description of the cytoplasmic calcium dynamics in the post synaptic site, including both calcium accumulation and calcium extrusion. Calcium accumulation in cytoplasm is mainly dependent on calcium influxes through NMDARs and calcium release from internal calcium store [for example, in endoplasmic reticulum (ER)].

### Calcium dynamics

Postsynaptic calcium dynamics in cytoplasm and in stores are expressed by Kusters et al. (2005):

(1) $$\begin{array}{l} Vol_{Cyt}\frac{d{\left[Ca^{2+}\right]}_{Cyt}}{dt}=A_{ER}\left(J_{IP_{3}R}+J_{IKER}-J_{SERCA}\right)\\ +\;\frac{10^{-6}}{Z_{ca}F}I_{NMDAR}-\frac{{\left[Ca^{2+}\right]}_{Cyt}-{\left[Ca^{2+}\right]}_{basal}}{\tau_{Ca}} \end{array}$$

(2) $$\begin{array}{l} Vol_{ER}\frac{d{\left[Ca^{2+}\right]}_{ER}}{dt}=A_{ER}\left(-J_{IP_{3}R}-J_{IKER}+J_{SERCA}\right) \end{array}$$

Here, *I*_*NMDAR*_ is the calcium current through NMDARs, *J*_*I*_*P*__3_*R*_ denotes calcium flux from the ER to the cytoplasmic space through IP_3_Rs, *J*_*SERCA*_ is the calcium flux pumped from the cytoplasmic space into the ER, *J*_*IKER*_ is the leak of calcium ions from the ER to cytoplasmic space, *Z*_*Ca*_ is calcium ion valence, *F* is Faraday's constant, *Vol*_*Cyt*_ is the volume of synapses, *Vol*_*ER*_ is the volume of stores, *A*_*ER*_ is the surface area of the store exposed in synapses, ${\left[Ca^{2+}\right]}_{ER}$ and ${\left[Ca^{2+}\right]}_{Cyt}$ are calcium concentrations of ER and cytoplasmic region, respectively, ${\left[Ca^{2+}\right]}_{basal}$ is the basal concentration of calcium in cytoplasmic region and the decay constant is τ_*Ca*_. The values of the different parameters in equations (1, 2) using in this paper are furnished in the Table 1.

**Table 1:** **Parameter values of calcium dynamics in cytoplasmic region**.

*Vol_Cyt_*	1 × 10^−12^	dm^3^
*Vol_ER_*	0.1 × 10^−12^	dm^3^
*A_ER_*	0.3 × 10^−7^	dm^2^
*Z_Ca_*	2	–
*F*	96,480	C/mol
[*Ca*^2+^]_*basal*_	0.1	μM
τ_*Ca*_	12	ms

*I*_*NMDAR*_ is given by Shouval et al. (2002)

(3) $$\begin{array}{l} I_{NMDAR}=P_{0}\;\cdot \;G_{NMDAR}\;\cdot \;\theta_{1}(t)\;\cdot \;\{I_{glut}^{f}\;\cdot \;\exp[(t_{glut}-t)/\tau_{glut}^{f}]\\ \;+\;I_{glut}^{s}\;\cdot \;\exp[(t_{glut}-t)/\tau_{glut}^{s}]\}\;\cdot \;H(V) \end{array}$$

where *V* is the postsynaptic potential and glutamate is released at a time *t* = *t*_*glut*_. Here *P*_0_ is the fraction of NMDARs that change to the open state after glutamate binding and *G*_*NMDAR*_ is the conductance of the open NMDARs from calcium ions. θ_1_ is a step function: θ_1_(*t*) = 1 when *t* ≥ *t*_*glut*_, and θ_1_(*t*) = 0 when *t* < *t*_*glut*_. Glutamate unbinding is characterized by two decay constants $\tau_{glut}^{f}$ and $\tau_{glut}^{s}$, and the fast and slow unbinding components $I_{glut}^{f}$ and $I_{glut}^{s}$. The term *H(V)* describes Mg^2+^ unblock due to changes in the postsynaptic potential and is modeled by

(4) $$\begin{array}{l} H\left(V\right)=\left(E_{Ca}-V\right)/\left(1.0+\frac{\left[Mg^{2+}\right]\cdot e^{-0.062V}}{3.57}\right), \end{array}$$

where *E*_*Ca*_ is the reversal potential of calcium, *V* is the postsynaptic membrane potential, and [*Mg*^2+^] is the external magnesium ion concentration. All parameter values used for simulation of *I*_*NMDAR*_ are listed in Table 2.

**Table 2:** **Parameter values of *I*_*NMDAR*_**.

*P_0_*	0.5	–
*G_NMDA_*	0.00035	μM/(ms·mV)
$I_{glut}^{f}$	0.5	–
$\tau_{glut}^{f}$	50	ms
$I_{glut}^{s}$	0.5	–
$\tau_{glut}^{s}$	200	ms
*E_Ca_*	130	mV
[*Mg*^2+^]	1.5	mM

The expressions for each calcium fluxes from the store are

(5) $$\begin{array}{l} J_{IP_{3}R}=f_{\infty }^{3}w^{3}K_{IP_{3}R}\left({\left[Ca^{2+}\right]}_{ER}-{\left[Ca^{2+}\right]}_{Cyt}\right), \end{array}$$

(6) $$\begin{array}{l} J_{IKER}=K_{IKER}\left({\left[Ca^{2+}\right]}_{ER}-{\left[Ca^{2+}\right]}_{Cyt}\right), \end{array}$$

(7) $$J_{SERCA}=J_{SERCA}^{max}\frac{{\left[Ca^{2+}\right]}_{Cyt^{2}}}{K_{SERCA}^{2}+{\left[Ca^{2+}\right]}_{Cyt^{2}}},$$

with

(8) $$\begin{array}{l} f_{\infty }=\frac{{\left[Ca^{2+}\right]}_{Cyt}}{K_{fIP_{3}}+{\left[Ca^{2+}\right]}_{Cyt}}. \end{array}$$

And *w* is the fraction of activated IP_3_Rs; its dynamics is modeled by

(9) $$\begin{array}{l} \frac{dw}{dt}=\frac{w_{\infty }-w}{\tau_{w}}, \end{array}$$

with

(10) $$\begin{array}{l} w_{\infty }=\frac{\frac{\left[IP_{3}\right]}{K_{wIP_{3}}+\;\left[IP_{3}\right]}}{\frac{\left[IP_{3}\right]}{K_{wIP_{3}}+\;\left[IP_{3}\right]}+K_{w\left(Ca\right)}{\left[Ca^{2+}\right]}_{Cyt}} \end{array}$$

(11) $$\begin{array}{l} \tau_{w}=\frac{a}{\frac{\left[IP_{3}\right]}{K_{wIP_{3}}+\;\left[IP_{3}\right]}+K_{w\left(Ca\right)}{\left[Ca^{2+}\right]}_{Cyt}}. \end{array}$$

*IP*_3_ is used to mimic activation of mGluRs, we describe the change in *IP*_3_ by production and degradation as follows (De Young and Keizer, 1992):

(12) $$\begin{array}{l} \frac{d\left[IP_{3}\right]}{dt}=-I_{r}\left[IP_{3}\right]+I_{P}f\left(t\right), \end{array}$$

Where [*IP*_3_] is concentration of *IP*_3_ in cytoplasm, *I*_*r*_ is the rate constant for degradation of *IP*_3_, *I*_*p*_ is the maximal production generated by each activation of mGluRs, and *f(t)* controls the timing of activation of mGluRs and is described by a step function: If *t*_*glut*_ + 2 ms > *t* > *t*_*glut*_, *f(t)* = 1; otherwise *f(t)* = 0. Details of the parameters are listed in Table 3.

**Table 3:** **Parameter values of calcium dynamics in stores and IP_3_ dynamics**.

*K*_*IP*_3_*R*_	6 × 10^−5^	dm/s
*K_IKER_*	0.002 × 10^−5^	dm/s
$J_{SERCA}^{max}$	8 × 10^−5^	μM/(s·dm^2^)
*K_SERCA_*	0.2	μM
*K*_*fIP*_3__	0.5	μM
*K*_*wIP*_3__	1.5	μM
*K*_*w*(*Ca*)_	0.5	μM^−1^
*I_r_*	0.5	s^−1^
*I_p_*	0.009	μM·s^−1^
*a*	20	s

Postsynaptic potential change has been modeled by (Shouval et al., 2002)

(13) $$\begin{array}{l} V=V_{rest}+EPSP, \end{array}$$

where *V*_*rest*_ is the resting membrane potential, *V*_*rest*_ = −65 mV. *EPSP* is the net depolarized voltage generated by binding glutamate to receptors in post synapse. The *EPSP* is:

(14) $$\begin{array}{l} EPSP=s\cdot \left[e^{\left(t_{glut}-\;t\right)/\tau_{1}}+e^{\left(t_{glut}-\;t\right)/\tau_{2}}\right], \end{array}$$

where *s* is the maximal potential of depolarization, *s* = 50 mV, τ_1_ and τ_2_ are membrane potential decay constants, τ_1_ = 50 ms and τ_2_ = 5 ms, and *t*_*glut*_ is the time that glutamate released.

### Pi and lisman model of the CaMKII/PP2A reaction network

In the present modeling, the CaMKII/PP2A reaction network exactly follows the work of Pi and Lisman (2008). The cytoplasmic calcium dynamics in the post synaptic site affects the rates of autocatalytic reactions that convert CaMKII and PP2A from their non-phosphorylated to phosphorylated forms. For CaMKII the concentration of the phosphorylated form, which is the active form, is denoted by K^*^, while for PP2A the non-phosphorylated form is active, and its concentration is denoted by P^*^. All equations describing the evolution of K^*^ and P^*^ were taken directly from Pi and Lisman (2008):

(15) $$\begin{array}{l} \frac{d}{dt}K^{*}=k_{1}\frac{K_{tot}-K^{*}}{K_{m1}+\left(K_{tot}-K^{*}\right)}K^{*}-k_{2}\frac{K^{*}}{K_{m2}+K^{*}}\left(P^{*}+P_{0}\right)\\ +\;k_{3}K_{0}+\;k_{4}\frac{{\left[Ca^{2+}\right]}_{Cyt}^{4}}{K_{m}^{4}+{\left[Ca^{2+}\right]}_{Cyt}^{4}}\left(K_{tot}-K^{*}\right) \end{array}$$

(16) $$\begin{array}{l} \frac{d}{dt}P^{*}=k_{11}\frac{P_{tot}-P^{*}}{K_{m11}+\left(P_{tot}-P^{*}\right)}P^{*}-k_{12}\frac{P^{*}}{K_{m12}+P^{*}}\left(K^{*}+K_{0}\right)\\ +\;k_{13}P_{0}+\;k_{14}\frac{{\left[Ca^{2+}\right]}_{Cyt}^{3}}{K_{m}^{3}+{\left[Ca^{2+}\right]}_{Cyt}^{3}}\left(P_{tot}-P^{*}\right) \end{array}$$

where *k*_1_, *k*_2_, *k*_3_, *k*_4_, *k*_11_, *k*_12_, *k*_13_, and *k*_14_ are rate constants for each reaction. *K*_*m*1_, *K*_*m*2_, *K*_*m*_, *K*_*m*11_, and *K*_*m*12_ are equilibrium constants for kinase and phosphatase. *K*_0_ and *P*_0_ indicate the basal concentration of active kinase and phosphatase. *K*_*tot*_ and *P*_*tot*_ indicate the total amount of kinase and phosphatase.

Dependence of AMPARs on K^*^ and P^*^ is described as:

(17) $$\begin{array}{l} \frac{d}{dt}A=\left(c_{1}K^{*}+c_{3}\right)\left(A_{tot}-A\right)\;-\;\left(c_{2}P^{*}+c_{4}\right)A, \end{array}$$

where *A* and *A*_*tot*_ indicate the AMPARs on the synaptic membrane and the total amount. *c*_1_ and *c*_2_ are scaling factors, *c*_3_ and *c*_4_ are rate constants for processes independent of kinase and phosphatase activities. All parameters of CaMKII/PP2A reaction network are listed in Table 4.

**Table 4:** **Parameter values of CaMKII/PP2A reaction network**.

*K_tot_*	20	μM
*P_tot_*	20	μM
*A_tot_*	1	–
*K*_0_	0.5	μM
*P*_0_	0.5	μM
*k*_1_	2	s^−1^
*k*_2_	15	s^−1^
*k*_3_	1	s^−1^
*k*_4_	120	s^−1^
*k*_11_	2	s^−1^
*k*_12_	15	s^−1^
*k*_13_	1	s^−1^
*k*_14_	80	s^−1^
*K_m_*	4	μM
*K*_*m*1_	10	μM
*K*_*m*2_	0.3	μM
*K*_*m*11_	10	μM
*K*_*m*12_	1	μM
*c*_1_	1	–
*c*_2_	1	–
*c*_3_	6	s^−1^
*c*_4_	8	s^−1^

### Numerical implementation

All the calculations were implemented in the C programming language, with the differential equations expressed as different equations, and solved by using the fourth order Runge–Kutta method in steps of 0.01 ms. According to the related experimental procedures, theta-burst stimulation (TBS) was used as triggers for elevation of calcium concentration in cytoplasm. One unit of TBS is consisted of 3 pulses (100 Hz) delivered at 200 ms intervals. For application of each specific units of TBS, the variation of cytoplasmic calcium concentration in the post-synaptic site, the values of concentration of active kinase and phosphatase, and synaptic efficacy were calculated as functions of time, respectively.

## Results

### Transitions of synaptic strength induced by CaMKII/PP2A network switching

**Figure 2:**
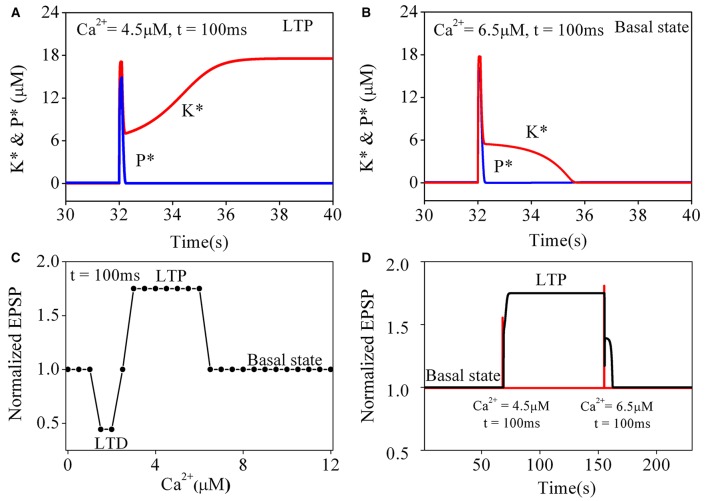
**Simulation results for original pi and lisman model of coupled kinase and phosphatase swithes in response to different simple calcium pulse**. K^*^ and P^*^ denote concentration of phosphorylated kinase and dephosphorylated phosphatase, respectively. **(A)** Changes of K^*^ and P^*^ in response to application of 4.5 μM calcium pulse (*P*_*Ca*_) for 100 ms. Before *P*_*Ca*_ stimulation, K^*^ and P^*^ both stay at basal level. During *P*_*Ca*_ application, K^*^ and P^*^ rise, and K^*^ is dominate. After *P*_*Ca*_ is over, P^*^ returns back to basal level while K^*^ stays at high level, which finally leads to LTP. **(B)** Changes of K^*^ and P^*^ in response to application of 6.5 μM calcium pulse for 100 ms. Both K^*^ and P^*^ are transiently increased by *P*_*Ca*_ and return back to basal level after Ca^2+^ is removed. Therefore, synaptic efficacy remains unchanged. Red line represents K^*^ and blue line for P^*^, respectively. **(C)** Dependence of synaptic efficacy on amplitudes of *P*_*Ca*_. As the level of calcium increase from moderate to high, synaptic efficacy changes from LTD to LTP, then return to basal state when amplitude of *P*_*Ca*_ increases further. **(D)** Reversals of LTP. LTP is induced by the first stimulation (4.5 μM, 100 ms) and reversed to basal level (depotentiation) by the second stimulation (6.5 μM, 100 ms). Changes in *P*_*Ca*_ is shown with red line and synaptic efficacy with black line, respectively.

To better understand the transition of synaptic strength, it is useful to consider how the original Pi and Lisman model switches in simpler situations. We begin with a study on the responses of the original CaMKII/PP2A network to a simple calcium pulse (*P*_*Ca*_) with different amplitude and the same duration (100 ms). Simulation results are shown in Figure 2. The results for *P*_*Ca*_ = 4.5 μM are consistent with those reported by Pi and Lisman (2008), changes of phosphatase and kinase activities are shown in Figure 2A. Both enzymes were nearly inactive in the basal state. During elevation of *P*_*Ca*_, the kinase and phosphatase become strongly activated. At the end of the induction period, *P*_*Ca*_ returns to the basal level, phosphatase activation returns back to its basal level subsequently but the kinase activation remains at a high level. In this way a LTP is induced.

However, further transient elevations of calcium concentration failed to induce a LTP state. The simulation result of changes in phosphatase and kinase activities to a transient increase of *P*_*Ca*_ to 6.5 μM is shown in Figure 2B. Similarly, two enzymes are inactive in the basal state. During *P*_*Ca*_ elevation, the kinase and phosphatase also become strongly activated. At the end of the induction period, *P*_*Ca*_ returns to its basal level and both phosphatase and kinase return to their basal states subsequently. So there is no sustained change in synaptic strength.

To further characterize the transitions between different states, we systematically varied amplitude of *P*_*Ca*_ with the duration fixed at 100 ms. For *P*_*Ca*_ between 0.1 and 1.3 μM, the system is stabled at basal state. For *P*_*Ca*_ in a range between 1.4 and 2.4 μM, the system is switched from the basal state to LTD states. For *P*_*Ca*_ between 3.0 μM and 6.2 μM, it is switched to LTP states. However, in conditions with *P*_*Ca*_ > 6.2 μM, neither LTP nor LTD is inducible (Figure 2C), the system returns to its basal state, suggesting that significant elevation of calcium concentration may depotentiate synaptic strength to basal levels.

Depotentiation from a LTP state was also tested by inputting a large *P*_*Ca*_ at a LTP level. As shown in Figure 2D, after a LTP is induced from the basal state by a proper *P*_*Ca*_ (4.5 μM for 100 ms), a large *P*_*Ca*_ (6.5 μM for 100 ms) reverses the potentiated state to its basal level, this mechanism of depotentiation is different from the ones induced by small elevations of *P*_*Ca*_ (Pi and Lisman, 2008), which induce LTD from a basal synaptic strength.

### The amount of TBS trains determines changes in cytoplasmic calcium concentration, K^*^, and P^*^

In order to directly simulate the experimental induction of depotentiation from a LTP state, we applied the induction procedure used in the experiments to the modified tristable system (including calcium dynamics) in simulation. A postsynaptic cytoplasmic calcium concentration dynamics produced by NMDARs activation and calcium release from internal calcium stores was considered and coupled in the original system. We then observed very different responses induced by application of different amount of TBS.

**Figure 3:**
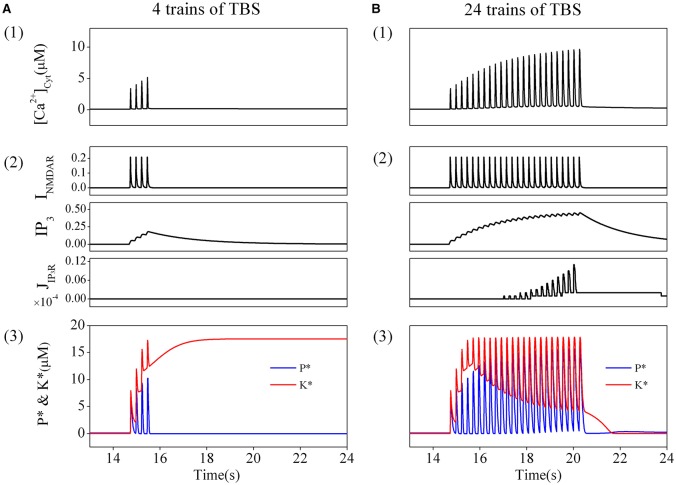
The amount of TBS trains determines the changes in cytoplasmic calcium concentration, K^*^, and P^*^. Cytoplasmic calcium concentration in post-synapse induced by a brief TBS (4 trains) **(A1)** and a prolonged TBS (24 trains) **(B1)**. Details of each calcium fluxes induced by the brief **(A2)** and the prolonged TBS **(B2)**. Enzyme activation induced by the brief TBS (4 trains) **(A3)** and the prolonged TBS **(B3)**. Before the brief TBS, both K^*^ and P^*^ are nearly inactive. During stimulation, both K^*^ and P^*^ are transiently increased. After calcium removal, K^*^ stays at high and P^*^ returns back to basal level. Therefore, LTP is induced. On the other hand, the prolonged TBS induces a depotentiation.

We first consider the responses to a brief TBS. A train of corresponding elevations of cytoplasmic calcium concentration is induced, and the amplitudes of these elevations are moderate (Figure 3A1). Details of the result are presented in Figure 3A2. Before application of the stimulation, no transmitters are released, thus NMDARs are closed, while the basal concentration of *IP*_3_ is very low. The IP_3_Rs are also closed, therefore, there are neither Ca^2+^ influx through NMDARs nor Ca^2+^ release through IP_3_Rs. During TBS delivery, NMDARs are activated to mediate a series of Ca^2+^ influx through NMDARs (Figure 3A2 top), while IP_3_Rs is still closed (Figure 3A2 bottom) as the concentration of *IP*_3_ is not high enough, although it shows growing pulsatile elevations (Figure 3A2 middle). The results show that, a comparatively high elevation in cytoplasmic calcium concentration is induced by brief TBS, which is mainly mediated by activation of NMDARs.

As shown in Figure 3A3, before elevation of cytoplasmic calcium concentration, both enzymes are nearly inactive in the basal state. During the elevation of cytoplasmic calcium concentration, kinase and phosphates become strongly activated. Then cytoplasmic calcium concentration decline to the basal level, phosphates returns back to the basal level subsequently, while the kinase still stays at the activated state. Therefore, synaptic efficacy is switched to a LTP state.

Then, we turn to consider the responses to a prolonged TBS. Correspondingly, a series of elevations of cytoplasmic calcium concentration is induced by this prolonged TBS, and the amplitudes of the elevations gradually increase to significantly high levels (Figure 3B1). We then analyzed in details the sources of Ca^2+^. Before the stimulation, both NMDARs and IP_3_Rs are closed and there is no Ca^2+^ influx. As this prolonged TBS is delivered, NMDARs are activated to mediate stronger Ca^2+^ influx (Figure 3B2 top), meanwhile, IP_3_Rs are gradually activated (Figure 3B2 bottom) as the concentration of *IP*_3_ is accumulated to a higher lever (Figure 3B2 middle). Therefore, a more significant elevation in cytoplasmic calcium concentration is eventually formed by activation of both NMDARs and IP_3_Rs.

Changes in responses of phosphates and kinase caused by this significant elevation in cytoplasmic calcium concentration are shown in Figure 3B3. Both enzymes are nearly inactive in the basal state. During this significant elevation in cytoplasmic calcium concentration, the kinase and phosphates become strongly activated. However, both of them return back to their basal states subsequently as cytoplasmic calcium concentration declines to basal levels. Therefore, synaptic efficacy remains at the basal state.

### Reversal of an established LTP

**Figure 4:**
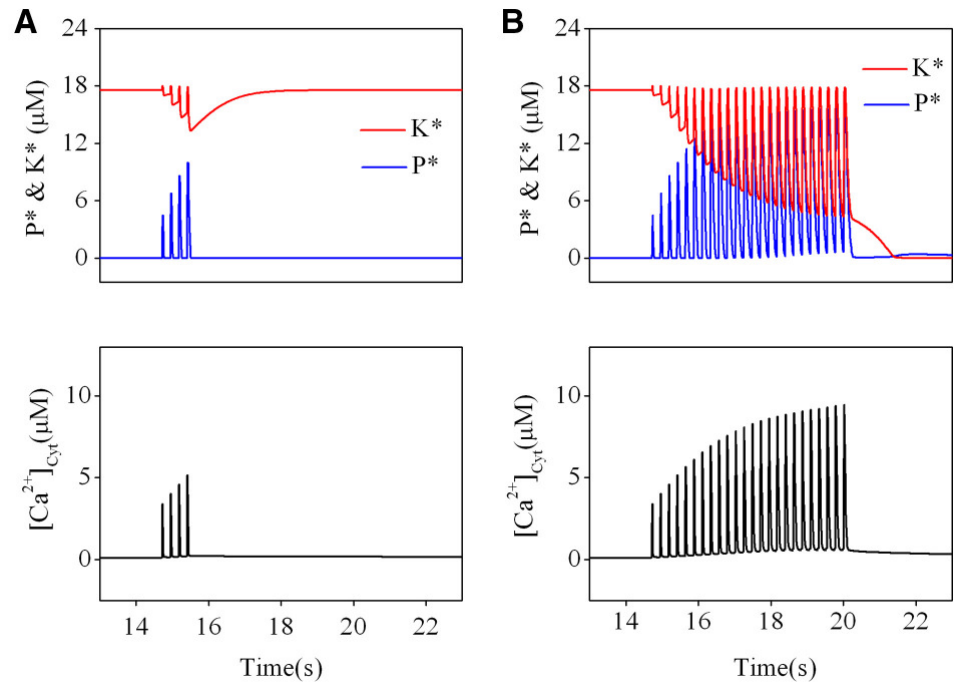
**Effect of different TBS on an established LTP**. Changes of kinase (K^*^) and phosphatase (P^*^) (upper) and calcium dynamics (bottom) induced by the brief TBS **(A)** and the prolonged TBS **(B)**.

Previous behavioral experiments have also shown that brief novelty acquisition was insufficient to reverse LTP, but a prolonged novelty exposure did reverse an established LTP (Qi et al., 2013). Similar phenomena were also observed in hippocampus slices by using electrical stimulations. In the CA1 region of hippocampus slices from young adult rats, TBS, which mimics a physiologically relevant frequency of neuronal activity exhibited in the hippocampus of behaving animals (Kandel and Spencer, 1961; Ranck, 1973; Bikbaev et al., 2008), induces long-lasting reversal of previously induced LTP (Barr et al., 1995). To test for whether a prolonged TBS can reverse LTP while a brief TBS cannot, we first simulated the effect of a brief TBS on established LTP. As shown in Figure 4A, activation of kinase was initially set at an activated state and phosphates at a basal state, corresponding to a LTP state of synaptic strength. When an elevation of cytoplasmic calcium concentration is induced by a brief TBS, kinase becomes slightly inactivated and phosphatase strongly activated. After cytoplasmic calcium concentration declines to the basal state, kinase returns to its initial activated state and phosphatase returns to its basal state. In other words, before and after brief TBS, activation levels of both kinase and phosphatase are unchanged. Hence, a brief TBS is not able to induce a transition from the established LTP state to the basal state.

Then, we simulated the effect of a prolonged TBS on an established LTP. The results are presented in Figure 4B. Before application of TBS, activation of kinase was still set at an activated state and phosphatase at a basal state. During a significant elevation by a prolonged TBS, kinase becomes strongly inactivated but phosphatase becomes strongly activated. When cytoplasmic calcium concentration declines to its basal level, activated forms of both kinase and phosphatase are switched to their basal states. The results clearly show that a prolonged TBS can reverse an established LTP by switching kinase from its activated state to its basal state, which is induced by a significant elevation of calcium concentration at the post synaptic site.

### Phase space analysis

**Figure 5:**
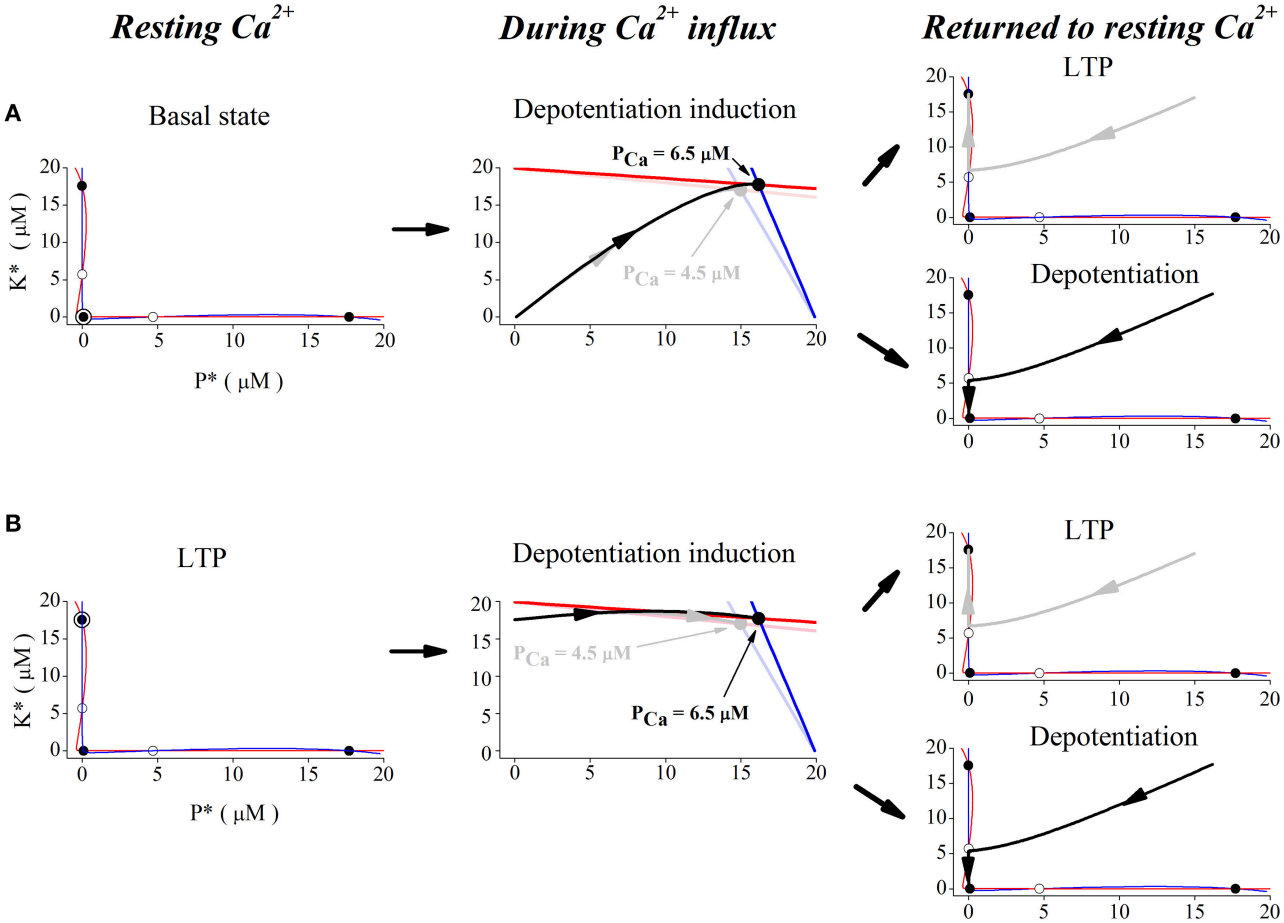
**Phase space analysis of the system's dynamics when initial state is at basal state (A) and at LTP (B), respectively**. Left panel, red and blue curves indicate nullclines for kinase and phosphatase, respectively. Nullclines at basal Ca^2+^ concentration (0.1 μM) create five steady-states. Open and filled circles denote unstable and stable steady-states, the filled circle with black open circle indicates the basal state **(A)** and LTP **(B)**, K^*^ and P^*^ denote concentration of active kinase and phosphatase, respectively. Middle panels, nullclines during Ca^2+^ influx. Ca^2+^ elevation deforms the nullclines to create one stable state. Red and blue lines indicate nullclines for K^*^ and P^*^ and black dot denotes the new stable state under 6.5 μM Ca^2+^ elevation. Light red and light blue lines indicate nullclines for K^*^ and P^*^ and gray dot denotes the new stable state under 4.5 μM Ca^2+^ elevation. The system becomes unstable and moves to the new stable states (gray trace for 4.5 μM and black trace for 6.5 μM) from basal state **(A)** and LTP **(B)**, respectively. Right panels, nullclines after Ca^2+^ removal. Nullclines form three stable states again in the basal Ca^2+^ level. The state moves to the closest stable state, LTP (gray trace, top), or basal state (black trace, bottom), respectively.

In order to show the dynamics of depotentiation graphically, the nullclines of CaMKII/PP2A network at different Ca^2+^ concentrations is given in Figure 5. Firstly, we studied the case with unchanged synaptic efficacy after action of Ca^2+^ pulses. In this case, the system stays at the basal state before application of Ca^2+^ pulses. At the basal Ca^2+^ level, nullclines (Figure 5A, left panel) intersects at five points; three are stable points (filled circles at top left, bottom left, and bottom right) and two are unstable (open circles). In the basal state, the system is stable at the bottom left point. During a high Ca^2+^ elevation, the nullclines are deformed to create only one stable state (Figure 5A, middle panel, light blue and light red lines, and gray dot), and the system finally stays at the new unique stable state (Figure 5A, middle panel, gray trace). After action of Ca^2+^ pulses, the three stable states restore, but the system stays at the top left stable point (LTP) (Figure 5A, top right panel). However, during a significant elevation of Ca^2+^ concentration, nullclines are deformed to create another unique stable state (Figure 5A, middle panel, blue and red lines, and black dot), and the system moves from this stable state (Figure 5A, middle panel, black trace) to and stays at the bottom left stable point (basal state), when the original three stable points restores after action of Ca^2+^ pulses (Figure 5A, bottom right panel).

Then, we studied reversal of the established LTP by application of Ca^2+^ pulses. At the LTP level, the system was stable at the top left stable point (Figure 5B, left panel). During action of high Ca^2+^ pulses, high Ca^2+^ elevations deform the nullclines to create one stable state (Figure 5B, middle panel, light blue and light red lines, and gray dot), and the system moves to this new stable state (Figure 5B, middle panel, gray trace). After action of Ca^2+^ pulses, nullclines restore the three stable states and the system returns to the top left stable point (LTP) (Figure 5B, top right panel). During action of significant Ca^2+^ pulses, large Ca^2+^ elevations deform nullclines to create the unique stable state (Figure 5B, middle panel, blue and red lines, and black dot), and the system moves to this new stable state (Figure 5B, middle panel, black trace). After action of Ca^2+^ pulses, nullclines restore the three stable states, but the system moves to and stays at the bottom left stable point (basal state) (Figure 5B, bottom right panel).

**Figure 6:**
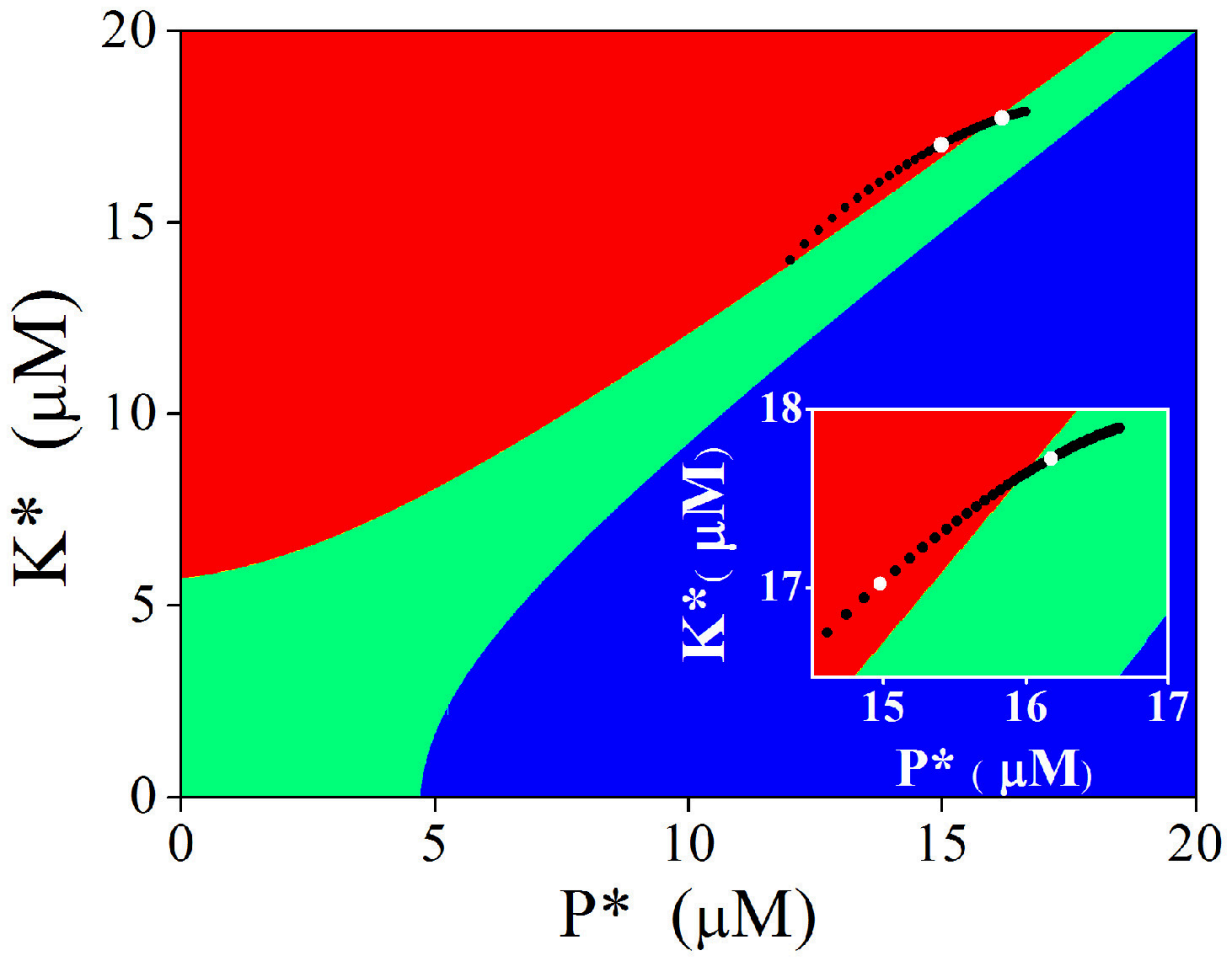
**Attractive basins with basal level Ca^2+^ and evolvement of the stable state induced by adjusting Ca^2+^ elevation**. Red region indicates the attractive basin of LTP, Green for basal state, and blue for LTD. Black dots indicate evolvement of the stable state. Two white dots represent the stable states induced by 4.5 μM **(bottom left)** and 6.5 μM **(top right)**, respectively (also shown in the insertion).

Attractive basins of each stable state were depicted in the K^*^ − P^*^ plane when calcium concentration was set at the basal level (Figure 6). We have known that nullclines form three stable states (filled circles at top left, bottom left, and bottom right of left panels in Figures 5A,B) at basal Ca^2+^ level. The K^*^ − P^*^ plane is then divided into three regions; points located within the red region will evolve to the stable point at top left (named LTP basin), those in the green region to the stable point at bottom left (named basal state basin), and those in the blue region to the one point at bottom right (named LTD basin), respectively.

Then we turned to observe the distribution of the stable points created by Ca^2+^ elevations in the K^*^ − P^*^ plane. As the amplitude of Ca^2+^ elevation increases from 3 to 10 μM gradually, the stable points induced by Ca^2+^ elevation moves from bottom left to top right, from the LTP basin to the basal state basin. The stable point induced by 4.5 μM Ca^2+^ elevation is in the LTP basin (white dot in left), while the stable point induced by 6.5 μM Ca^2+^ elevation is in the basal state basin (white dot in right, graph inserted in Figure 6). After the action of Ca^2+^ elevation, nullclines restore the three stable states, and the system will evolve from its new initial position (the position determined by Ca^2+^ elevation) to one of the three stable states, according to the location of this initial position in the K^*^ − P^*^ plane. The new initial position determined by high Ca^2+^ elevation is in the LTP basin and the system evolves to LTP. The new initial position determined by significant Ca^2+^ elevation is in the basal state basin, therefore, the system evolves to the basal state and can manifest in this way the depotentiation from a LTP state. Just as in the original CaMKII/PP2A network model, Ca^2+^ elevation is still the primary controller, which controls the synaptic strength through activation of kinase and phosphatase. In experimentations synaptic strength is not directly manipulated by Ca^2+^ elevation but by presynaptic simulation, including TBS. The above analysis provides a link between experiments and the generic dynamics revealed by the tristable system (Pi and Lisman, 2008).

## Discussion

Although there has been substantial investigation on how a tristable system composed of coupled kinase and phosphatase switches could account for stable transition of synaptic strength from basal state to both LTP and LTD, depotentiation from LTP to basal state has not been theoretically studied. Based on a series of experimental observation, the present work introduced two Ca^2+^ resources with different temporal dynamics, mediated by influx through NMDARs and release from internal calcium store, respectively, in the generic tristable system and further show that significant elevation of cytoplasmic calcium concentration may dynamically switch activation of both kinase and phosphatase to their basal states, thereby depotentiate the synaptic strength. The generation of this significant elevation of cytoplasmic calcium concentration depends on the length of stimulation, a prolonged stimulation activates both NMDARs and internal Ca^2+^ stores and then produces a significant Ca^2+^ elevation, while a brief stimulation only activates NMDARs and induces a high Ca^2+^ elevation. When initial condition of synaptic strength is at basal state, a prolonged TBS dynamically firstly induces a high Ca^2+^ elevation which might result in LTP, and then induces a large Ca^2+^ elevation and depotentiates the possible LTP.

Involvement of internal calcium store is required for induction of depotentiation. Both experiments and simulations have shown that experience exposure or stimulation protocol elevating cytoplasmic calcium concentration by activating cellular membrane calcium channels can switch the synaptic strength from basal level to LTP. We have shown in this paper that repeated experience exposure or prolonged stimulation could further activate postsynaptic group I mGluRs, produce more *IP*_3_ to activate IP_3_Rs on endoplasmic reticulum, finally lead to calcium release from internal calcium store. Relatively slow dynamics of this process requires repeated experience or prolonged stimulation. Recent experiment results show that in synapses containing endoplasmic reticulum, presynaptic stimulation induced two successive elevation peaks of calcium concentration: The first one is mediated by NMDARs and the second by IP_3_Rs activation, respectively (Holbro et al., 2009; Sheridan et al., 2014). These experiments support the simulation results of the present paper, not only by showing the necessity of a significant calcium elevation, but also by an equivalent time course for induction of depotentiation. The results in our modeling also suggest that depotentiation should have a threshold-like effect with calcium concentration. The prediction of this threshold and its changes with changes of internal calcium store needs to be demonstrated in further experiments.

In an interesting experiment, mutant mice lacking IP_3_Rs display a significantly greater magnitude of LTP induced by tetanus stimulation (Fujii et al., 2000). A similar mutant strain lacking ryanodine receptor type 3 (RYR3), another type of calcium channel on internal calcium store, exhibits in hippocampus CA1 pyramidal neurons facilitated LTP as well (Futatsugi et al., 1999). These results are controversial to some expectations for release of calcium from internal store facilitates LTP. Our simulation work could provide an explanation for these experiments, by indicating that lack of IP_3_Rs or RYR3 may impair calcium release, and thus prevent depotentioation, finally facilitate LTP.

Compared with the calcium elevation inducing LTP, more significant and prolonged elevation of cytoplasmic calcium concentration is required for depotentiation induction. It has been proposed that moderate elevation in [Ca^2+^]_*cyt*_ that is produced during LTD induction may preferentially activate calcinerin, the Ca^2+^/calmodulin (CaM)-dependent protein phosphatase. Calcineurin then can induce inhibition of protein 1 (I-1), the endogenous inhibitor of serine/threonine protein phosphatase-1 (PP1). This allows the activation of PP1 (Oliver and Shenolikar, 1998). PP1 dephosphorylates CaMKII and other proteins (Shields et al., 1985), including glutamate receptors, to promote LTD. However, LTP-inducing stimuli are associated with higher increase in cytoplasmic calcium concentration. This can activate CaMKII, which plays a key role in LTP induction through phosphorylation of the GluR1 subunit of AMPARs (Cormier et al., 2001). In addition, high elevation of cytoplasmic calcium concentration can activate PKA, PKA can phosphorylate and activate I-1, which then can inhibit PP1 (Barria et al., 1997). Thus, LTP is induced and LTD is prevented.

PKA, which negatively regulates PP1, is modulated by the activation of Ca^2+^/CaM-dependent adenylyl cyclases (AC) (Piascik et al., 1980; Potter et al., 1980; Ahlijanian and Cooper, 1988). And the activity of AC, which shows a bell-shaped activity curve relative to increasing calcium concentrations (Piascik et al., 1980; Potter et al., 1980; Ahlijanian and Cooper, 1988), may be decreased by large rise in cytoplasmic calcium concentration induced by prolonged TBS. The resulting low PKA activity is accompanied by a disinhibition of PP1, which inactivates CaMKII, leading occurrence of depotentiation (Lisman, 1989).

When certain memory becomes obsolete, effective extinction of the previously established memory is essential for animals to adapt to the changing environment. An exposure-based therapy for human traumatic fear memories has long been established (McNally, 2007). Fear memories present as defensive reactions to the neutral cue for a period of up to many months after learning. However, when conditioned subjects repeatedly encounter a neutral cue without a reinforcing unpleasant event, a large rise in cytoplasmic calcium concentration decreases kinase activity and subsequently induces depotentiation by AMPA receptors endocytosis (Clem and Huganir, 2010). The present simulation results may account for the mechanism of these behavioral investigations.

It has been studied and clinically applied that agonist of NMDA receptors, D-cycloserine, could facilitate fear extinction when given systematically or locally into the amygdale (Davis, 2010). Targeting mGluRs and calcium pump may provide other mechanistic insights into potential treatments for fear extinction. Moreover, significant elevation of cytoplasmic calcium concentration resulting in depotentiation is achieved by enzyme reactions, hence modulations on the activities of enzymes become to the second kind of approaches. It is observed that ACs is required for spatial memory extinction (Zhang et al., 2011) and mice over-expressing type 1 ACs show enhancing spatial memory extinction (Zhang and Wang, 2013). Overall, the present findings uncover a potentially important time- and state-dependent mechanism of NMDAR- and mGluR-dependent depotentiation. Furthermore, the engagement of such depotentiation may be involved in the activity-dependent erasure of recently stored information in the hippocampus (Zhang et al., 2011; Wang and Zhang, 2012; Zhang and Wang, 2013), showing strong parallels with similar mechanisms engaged in the amygdala (Kim et al., 2007).

## Author contributions

MC prepared the methods of feature construction and conducted the experiments. WR and MC prepared the manuscript. WR and XW supervised all aspects of the work. All authors discussed the results and commented on the manuscript.

## Funding

This work was supported by the Fundamental Research Funds for the Central Universities of China (No. GK201503027 and GK201601001) and the National Natural Science Foundation of China (No. 11375109).

### Conflict of interest statement

The authors declare that the research was conducted in the absence of any commercial or financial relationships that could be construed as a potential conflict of interest.
